# Can health care databases be used to identify incident cases of osteonecrosis?

**DOI:** 10.1186/ar2731

**Published:** 2009-06-17

**Authors:** Steven C Vlad, David T Felson, Donald R Miller

**Affiliations:** 1 Clinical Epidemiology Research and Training Unit, Boston University School of Medicine, 650 Albany Street, X200, Boston, MA 02118, USA; 2 Division of Rheumatology, Boston University School of Medicine, 72 East Concord Street, E-5, Boston, MA 02118, USA; 3 Center for Health Quality, Outcomes, and Economic Research, Edith Nourse Rogers Memorial Veterans Hospital, Building 70, 200 Springs Road, Bedford, MA 01730, USA; 4 Department of Health Policy & Management, Boston University School of Public Health, 715 Albany Street, Talbot Building, Boston, MA 02118, USA

## Abstract

**Introduction:**

Osteonecrosis (ON) is a rare disease associated with alcohol and glucocorticoid use. Identifying additional risk factors is difficult as the number of cases at any single center is small. We investigated whether data available in large health care databases can be used to identify incident ON cases.

**Methods:**

Using data from the Boston Veterans Affairs Healthcare system, we identified potential cases of ON. These records, including available radiographs and reports, were reviewed. Using published criteria, we evaluated whether the subjects had confirmed ON (radiographs/reports met criteria), incident ON (onset of symptoms within 6 months of first code), or prevalent ON (onset more than 6 months prior to first code or onset could not be determined). We tested different definitions for incident ON using information derived from administrative data. These were compared to the 'gold standard' (record review) and positive predictive values (PPVs) were derived. Since PPVs for incident cases were low, we found the number of incident cases expected for every 1,000 potential cases identified, using the definitions as an initial screening tool to reduce the number of medical records that required examination.

**Results:**

We identified 87 potential cases. No case of jaw ON was identified. Only 15 (17%) incident cases of ON were identified. PPVs never exceed 50% for incident ON. However, if we used the definition '(at least 1 inpatient ON code) and (no prior codes for osteoarthritis)' as an initial screen, then for every 1,000 records, we would need to review only 150 to find 69 incident cases.

**Conclusions:**

Though the precise PPVs we found may not be generalizable to other databases, we believe that administrative data alone should not be used to identify incident cases of ON without confirming the diagnosis through a review of medical records. By applying the above definition, the number of records requiring review can be markedly reduced. This method can be used to find cases for valid case-control studies of risk factors for ON.

## Introduction

Osteonecrosis (ON) is a condition in which the vascular supply to an area of bone is interrupted, thereby causing its death. This leads to pain, collapse of the affected bone, destruction of the contiguous joint (osteoarthritis, or OA), and significant functional disability. Though uncommon, it is often devastating to young and old alike. Treatment is limited to surgery (often replacement of the affected joint), and strategies to prevent ON are badly needed.

Some established risk factors for ON have been identified and include trauma, alcoholism, and glucocorticoid (GC) use as well as conditions such as Gaucher disease, sickle cell disease, and autoimmune diseases [1]. Most of these risk factors have been identified through case reports and case series. While it is thought that around 60% of non-traumatic ON cases are accounted for by alcohol or GC use, the remainder are of unknown cause [2]. All risk factors appear to directly or indirectly elevate the risk of either thrombosis or embolism – whether by thrombosis or lipids – in the vessels supplying the affected bone [3]. Recently, uncontrolled hyperlipidemia was suggested to increase risk [4] and the putative link between bisphosphonate use and jaw ON [5] has been well publicized.

Finding new factors that could predispose a patient to or prevent ON, such as medications, can be difficult due to its rarity: it is thought that only 15,000 cases per year occur in the US [6], although this is far from clear. Any one center is unlikely to see enough cases to perform a valid study of risks with an adequate control group. Yet studies of this kind are necessary to better identify risk factors for ON and identify persons who might be at high risk.

The use of health care databases such as those maintained by large health care systems may allow the study of adequate numbers of persons with ON, permitting investigators to perform valid studies to identify and quantify the effect of new risk factors. This is especially true of pharmacologic risk factors as many of these large databases have detailed associated prescribing or dispensing information or both.

In the US, these datasets often identify medical conditions through the use of ICD-9-CM (International Statistical Classification of Diseases and Related Health Problems, Ninth Revision, Clinical Modification) codes, assigned by health practitioners at the time of patient visits. Valid studies of ON require a better understanding of how well these codes identify ON and whether other data can be used to better define ON cases.

The primary goal of this study was to find a method to identify cases of incident ON using data from a large health care database. We conducted our investigation by using a number of ICD-9 codes as well as other routinely available administrative data. Given the rapid development of pain in many persons with incident ON, we expected to find an algorithm with a high positive predictive value (PPV). Should our primary goal have given unacceptably low PPVs, a secondary goal was to find a method to efficiently identify incident ON using a combination of administrative data and chart review.

## Materials and methods

### Overview

We tested two possible strategies for finding incident ON cases in a hypothetical case-control study nested within a large health care cohort. The first would use ICD-9 codes and other administrative data likely to be available to find cases, but cases would not be otherwise verified. A sufficiently high PPV for a given algorithm would suggest that it could be used to identify cases without further need for verification. The second strategy would use the same administrative data to identify potential cases, but the medical records of these potential cases would then be reviewed to confirm which were actual incident cases. This strategy is less efficient than the first but would yield few if any false-positive incident ON cases (high specificity) and could be necessary if no algorithm with a high PPV could be identified. These confirmed cases could be used as the basis of well-designed case-control studies, which can yield valid results comparable to those of cohort studies [7].

The protocol for this study complied with the Declaration of Helsinki and was approved by the institutional review boards of the Veterans Affairs (VA) Boston Healthcare System, Edith Nourse Rogers Memorial Veterans Hospital, and the Boston University Medical Center. The requirement for informed consent was waived by all of these bodies.

### Data source and identification of potential cases

We used data from the VA Boston Healthcare System (VISN1). From 1 October 1998 through 30 September 2006, we attempted to identify veterans seen at VA Boston who were seen for ON; 76,155 veterans were seen in the inpatient or outpatient setting at least once during this period. We used the following ICD-9 codes to identify patients who could have had ON: 733.4× (ON), 732.7 (osteochondritis dissecans), 732.5 (Freiberg infarction), 526.89 (osteoradionecrosis of the jaw), and 526.4 (osteomyelitis or osteitis of the jaw). The second and third codes are conditions related to or easily confused with ON. The fourth and fifth were used as there was no code for ON of the jaw until recently (733.45 since 1 October 2007 [8]). We called all identified patients 'potential cases' of ON and reviewed all of their medical records. Note that the last digit (x) of the ON code (733.4×) is meant to identify the anatomic site of ON, but this digit was used inconsistently in our database; we therefore did not try to classify site based on the ICD-9 code.

### Case confirmation

VA has a standardized nationwide medical record system that incorporates almost complete records of all patient visits, including clinic notes, discharge summaries, radiology reports, and actual radiographs. The complete medical record of each veteran can be accessed from any one clinical location, allowing a complete record of their VA-provided health care, even as the patient moves from site to site in the US.

A board-certified rheumatologist (SCV) reviewed all of the available clinical notes, radiology reports, and actual radiographs of each potential case. Using the radiographic criteria established by Sugano and colleagues [9], we first confirmed that ON was present. Of these criteria, we ignored the requirement for no joint space narrowing or acetabular malformation as some late-stage cases, which clearly started as ON (for example, magnetic resonance imaging band pattern), had progressed to severe OA with attendant narrowing and remodeling of the acetabulum. We also did not use the histologic criterion as histology was usually unavailable. If radiographs were unavailable, a description in the radiology report or clinic notes consistent with these criteria (for example, 'a subchondral lucency') was sufficient to establish the diagnosis.

If ON was present, the case was called 'confirmed' and we examined the clinic notes further to find a description of when the symptoms leading to diagnosis began. If symptoms appeared to begin within 6 months of the first recorded ON code, the case was called 'incident'. If symptoms began more than 6 months prior to the first code or if the time of symptom onset could not be determined, the case was called 'prevalent'.

After initially reviewing the patient records, we decided to use 6 months to define incident ON. We did this for two reasons: (a) sometimes a patient had had symptoms for some months prior to orthopedic referral, at which time a definitive diagnosis was made and the code appeared in the chart; (b) although a diagnosis of ON was sometimes suspected by the referring physician and work-up begun, it was often not coded until the orthopedic consultant saw the patient. Six months appeared to us to offer the best balance between including long-standing cases for which it would be difficult to assess prior risk factors and excluding too many new cases that would otherwise be excluded with a too-stringent definition of time between initial symptoms and codes.

### Defining osteonecrosis using administrative data

Because detailed patient data such as those we used for our gold standards are unlikely to be present in large health care databases, we used VA administrative data (that is, data obtained from the administrative records of patient visits as opposed to data extracted directly from the medical records) to test different definitions of ON against the gold standard definitions. Elements from these administrative data included the above codes, dates when codes were assigned, the source of each code (primary care clinic [PCC] versus non-PCC, inpatient visit versus outpatient visit), and the numbers of each code. After the initial review of the records suggested that some ON cases that were first coded around the time of hip replacement had radiographs suggesting long-standing OA as a result of much earlier hip ON, we added definitions including OA. We hoped that this might permit us to identify non-incident ('prevalent') ON and exclude such persons from our search for incident cases. This review also suggested that only the specific ON code (733.4×) was of value in identifying cases; thus, no definition uses any other code for ON (see Results).

Using these elements, we created definitions designed to identify incident ON in administrative databases. These definitions included (a) one or more 733.4× ON codes anywhere in the record (that is, the same definition as that used to identify potential cases), (b) two or more ON codes anywhere in the record, (c) one or more ON codes from an inpatient visit, (d) one or more ON codes from a non-PCC (that is, specialty clinic) visit, (e) (one or more ON codes from an inpatient visit) or (one or more codes from a non-PCC visit), (f) (one or more ON codes from an inpatient visit) or (two or more ON codes, with at least one from a non-PCC visit), (g) (one or more ON codes) and (no prior codes for OA), and (h) (one or more ON codes from an inpatient visit) and (no prior codes for OA).

### Testing administrative definitions of osteonecrosis

We first tested each administratively derived definition of ON against the gold standards (strategy 1); that is, we identified all potential cases that would have been found using the administrative definitions, and of these, we found the number that were prevalent cases and the number that were incident cases. In so doing, we found the PPV of each administrative definition for both confirmed (incident + prevalent) and incident (number of cases/number of potential cases using that definition) ON.

We then determined, for each definition, how many cases we could expect to find after reviewing all of the records of the potential cases for that definition (strategy 2). First, we estimated the number of potential cases that would be identified using each administratively defined definition assuming that 1,000 potential cases would be found by the most liberal definition (definition 1). This is 1,000 times the number of potential cases for each definition divided by the number of potential cases for definition 1. Then we found how many of these cases could be expected to be incident ON after reviewing them. This is the number of potential cases for that definition times PPV. Results are expressed as numbers per 1,000 potential cases that would have been found using definition 1.

## Results

We identified and reviewed 94 potential ON cases. None of these was identified using the codes for osteochondritis dissecans, jaw osteomyelitis/osteitis, or Freiberg infarction. Seven were identified using the code for jaw osteoradionecrosis; all seven of these cases were associated with prior radiation therapy for head/neck malignancies and were not of interest. The remaining 87 potential cases were identified using the ON code (733.4×). We therefore decided that only the ON code should be used to identify cases; thus, these 87 potential cases are the denominator of all subsequent calculations.

One electronic record could not be retrieved for review and it was classified as a non-case. The ages of potential cases ranged from 21 to 92 years, and 92% were men (Table 1). Of these 87 potential cases, 81 were confirmed cases: 66 (76%) were prevalent cases, and 15 (17%) were incident cases (symptoms began within 6 months of the first code). The remaining 6 cases did not meet our criteria for ON. Seventy-one confirmed cases occurred in the femoral head (88%), with the remainder occurring in the humerus (4), talus (2), carpal navicular (2), and other sites in the foot/ankle (2). Fourteen of fifteen incident cases were in the femoral head (92%), with only one other incident case in the foot or ankle.

**Table 1:** Features of potential cases^a ^of osteonecrosis

Number of potential cases	87
Age, years	21 to 92
Women	7 (8%)
Sites (confirmed cases^b^)	81 (85% of potential cases)
Femoral head	71 (88%)
Humerus	4 (5%)
Talus	2 (2%)
Carpal navicular	2 (2%)
Other foot/ankle	2 (2%)
Sites (incident cases^c^)	15
Femoral head	14 (93%)
Humerus	0
Talus	0
Carpal navicular	0
Other foot/ankle	1 (7%)

^a^Potential cases were identified by the presence of at least one ICD-9 (International Statistical Classification of Diseases and Related Health Problems, Ninth Revision) code for osteonecrosis (733.4×) in the medical record. ^b^Confirmed cases were those in which osteonecrosis was present after reviewing the medical record. ^c^Incident cases were those in which symptoms were noted in the medical record to have begun within 6 months of the first code.

The PPVs for confirmed ON derived using the administrative definitions were generally high, ranging from 80% to 100% (Table 2). However, we noted that a large number of confirmed cases of ON of the femoral head were of late stage (Steinberg stage V or VI) [10] with severe OA, suggesting that they had been present for some time; this impression was confirmed by review of these medical records. Many of these cases had been coded as hip OA for some time prior to the appearance of the first ON code, suggesting that ON either had been unrecognized to that point or had simply been coded as OA since it had progressed to OA. In these cases, the first ON code sometimes appeared close to or at admission for hip replacement, suggesting that an orthopedic surgeon recognized the underlying reason for hip OA. Recognition of this informed our decision to test administrative definitions of ON which excluded prior diagnoses of OA.

**Table 2:** Main results for the 87 identified potential cases of osteonecrosis

Definition using administrative data	Number of records meeting definition (percentage)	PPV for confirmed ON (prevalent and incident)	PPV for incident ON	Number of records needing review for every 1,000 with any ON code	Number of incident ON cases expected after review
At least 1 ON code	87 (100%)	90%	17%	1,000	170
More than 1 ON code	85 (98%)	92%	18%	980	176
At least 1 inpatient ON code	47 (54%)	100%	23%	540	124
(At least 1 ON code) and (no prior codes for OA)	25 (29%)	76%	24%	290	70
(At least 1 inpatient ON code) and (no prior codes for OA)	13 (15%)	100%	46%	150	69

OA, osteoarthritis; ON, osteonecrosis; PPV, positive predictive value.

The PPVs for incident ON ranged from 17% to 46% (Table 2). The most general definition, 'one or more ON codes', identified 87 potential cases, of which 15 were incident ON. The definition with the best PPV (46%) was '(one or more ON codes from an inpatient visit) and (no prior codes for OA)', which identified 13 potential cases, of which 6 were incident ON. Because the PPVs for all of our definitions were low, we decided that confirmation of individual cases was likely to be necessary (strategy 2). With this strategy, for every 1,000 potential cases identified using definition 1, all would have to be reviewed to identify 170 cases of incident ON (since PPV was 17%). However, by using the definition with the best PPV (definition 8), for every 1,000 potential cases identified using the most sensitive definition, only 150 would need to be reviewed. This review would be expected to identify 69 incident cases but miss the remaining 101. Other definitions could be used to find more cases but at the expense of requiring review of more case records.

## Discussion

The growing presence of large health care databases offers unique opportunities to study risk factors for rare diseases. Many of these databases now include clinical data from hundreds of thousands if not millions of persons, offering the opportunity to identify a large number of diseased subjects, larger than can reasonably be expected to be seen at any one center.

ON provides a good example of such a disease. A rare condition, ON has an estimated rate of occurrence of only 15,000 cases per year in the US (good population-based estimates are lacking, however). Prior studies of risk factors are almost exclusively based on case series from a single center or a few centers, and although some of these are quite large and have yielded valuable information, relatively small numbers and lack of control groups limit the study of risk factors and limit appreciation of their relative strengths, including any which may pose even higher risks in persons who are already at high risk of ON (for example, elevated lipids in persons starting GCs). Also limiting is the lack of prospectively collected data on features such as medication use, comorbid illnesses, and other risk factors that often have been collected only at the time of diagnosis, subjecting such studies to recall bias.

Using large collections of health care data, collected in real time as patients are seen, offers an appealing method of dealing with some of these problems. However, as these are not primary research databases in which standard protocols are used to collect data, they are subject to limitations. In the case of ON, one of these limitations is that of establishing whether what appears to be a new case of ON as defined by a diagnosis code in the medical record is actually a true incident case. Establishing incidence is important in risk factor studies as we are more likely to identify a causal relationship between an exposure and a disease rather than an association in which cause and effect may not be immediately discernible.

We have shown that finding incident ON cases using only administrative data is problematic. Although ICD-9 codes are relatively specific for what we called 'confirmed' cases of ON, which include long-standing cases, a new code does not necessarily indicate an incident case. Does this mean we should give up hope of using these databases to study ON and other rare diseases? Not necessarily. First, prevalence studies can still yield valuable information about possible disease risks, although follow-up studies may then be required for confirmation. Second, using ICD-9 codes as a 'first pass' method of identifying possible cases and subsequently screening medical records to confirm incident cases, though more labor-intensive, could be of value. These confirmed cases could be used as the basis of well-designed case-control studies, which can yield valid results comparable to those of cohort studies [7]. We have shown that this method is probably feasible for identifying incident ON cases, yielding about one case for every two records reviewed using our best definition. Although this method will not identify all cases, this is not necessarily a threat to the validity of a well-designed case-control study as long as non-identified cases are not systematically different from identified cases.

Another benefit to reviewing all potential cases is that times between incidence of symptoms and appearance of the code can be assessed for each patient. This would allow investigators to use a date that better reflects the true incidence date of ON which in turn would allow a more precise assessment of the exposure-disease relationship.

Although many studies have 'validated' ICD-9 codes in large databases, we were unable to find any that addressed the issue of identifying incident versus prevalent cases. Often, studies are implicitly validating diagnosis codes for incident disease. This is the case for studies of myocardial infarction, stroke, and gastrointestinal hemorrhage, for instance [11-15]. In each of these, the condition is acute, has a very rapid onset, and is potentially life-threatening. Presentation to a hospital is assumed to occur quickly and require a subsequent inpatient stay. Therefore, using discharge summaries to identify incident cases seems very reasonable.

Validation studies for chronic diseases have also been published [16,17]. These generally, though implicitly, are studies of disease prevalence rather than incidence. Studies based on these codes are thus really risk association studies and could confuse cause and effect. The use of algorithms to identify incident disease could help to establish causal relationships in these studies. This is what we have attempted to do in the case of ON.

For chronic diseases such as ON, which ideally (though maybe not in fact) have a well-defined time of onset, differentiating between incidence and prevalence may be much easier than for chronic diseases such as chronic obstructive lung disease, in which defining the time of incidence is very difficult if not impossible. Using large health care databases to identify risk factors for many chronic diseases may therefore be formidably challenging.

Limitations of this study include the fact that precise PPVs that we derived are unlikely to be applicable to other databases or indeed to other VA centers; we would have needed to test our algorithms using other, independent patient samples to establish their generalizability. However, we think it is generally probable that diagnostic codes alone are not sufficiently predictive to identify incident ON. Diagnosis codes are not designed to differentiate incident from prevalent disease, and because most US centers and data sources use ICD-9 codes, it is likely that other centers and health care systems would find similar difficulties in identifying incident ON cases. Even high-volume centers are likely to see patients well after their initial symptoms occurred. Ideally, we would have determined the sensitivity and specificity of our definitions in addition to the PPV. Unfortunately, as with most studies with similar goals, this was not possible.

## Conclusions

ICD-9 codes for ON are not sufficiently predictive, in themselves, to be used to define incident ON in large pharmacoepidemiology studies. Review of medical records to confirm the diagnosis and determine the date of onset is recommended. However, simple algorithms can be used to alleviate the workload associated with chart review and yield cases for valid case-control studies.

## Abbreviations

GC: glucocorticoid; ICD-9 (CM): International Statistical Classification of Diseases and Related Health Problems, Ninth Revision (Clinical Modification); OA: osteoarthritis; ON: osteonecrosis; PCC: primary care clinic; PPV: positive predictive value; VA: Veterans Affairs.

## Competing interests

The authors declare that they have no competing interests.

## Authors' contributions

SCV designed the study, performed all chart reviews, performed all data analyses, and prepared the original and subsequent drafts of the manuscript. DTF and DRM contributed to the study design, interpretation of the data, and revision of the manuscript. All authors read and approved the final manuscript.
